# Enhancing the Efficiency of Color Conversion Micro-LED Display with a Patterned Cholesteric Liquid Crystal Polymer Film

**DOI:** 10.3390/nano10122430

**Published:** 2020-12-05

**Authors:** En-Lin Hsiang, Yannanqi Li, Ziqian He, Tao Zhan, Caicai Zhang, Yi-Fen Lan, Yajie Dong, Shin-Tson Wu

**Affiliations:** 1 College of Optics and Photonics, University of Central Florida, Orlando, FL 32816, USA; enlinhsiang@knights.ucf.edu (E.-L.H.); yananqili@knights.ucf.edu (Y.L.); zhe@knights.ucf.edu (Z.H.); tao.zhan@knights.ucf.edu (T.Z.); Yajie.Dong@ucf.edu (Y.D.); 2 Department of Materials Science & Engineering, University of Central Florida, Orlando, FL 32816, USA; cczhang@knights.ucf.edu; 3 NanoScience Technology Center, University of Central Florida, Orlando, FL 32826, USA; 4 AU Optronics Corp., Hsinchu Science Park, Hsinchu 300, Taiwan; Even.YF.Lan@auo.com

**Keywords:** micro-LED display, perovskite nanocrystals, cholesteric liquid crystal, wide color gamut, color conversion efficiency

## Abstract

Color-converted micro-light-emitting diode (micro-LED) displays with wide color gamut, high ambient contrast ratio, and fast response time are emerging as a potentially disruptive technology. However, due to limited optical density and thickness of the color-conversion film, the blue light leakage and low color-conversion efficiency still hinder their widespread applications. In this paper, we demonstrate a patterned cholesteric liquid crystal (CLC) polymer film with two special optical functionalities. On the green and red sub-pixels, the corresponding planar CLC texture acts as a distributed Bragg reflector for the blue light, which in turn improves the color conversion efficiency and expands the color gamut. On the blue sub-pixels, the corresponding focal-conic CLC texture acts as light scattering medium, which helps to reduce the angular color shift. Further analysis reveals that the patterned CLC film can alleviate the crosstalk between green and blue color filters. Therefore, compared to the display system without such a CLC film, our proposed device structure increases the color conversion efficiency by 143% (at ~90% Rec. 2020) and reduces average angular color shift Δu’v’ from 0.03 to 0.018 at the viewing angle with the most severe color shift. Such a patterned CLC film is applicable to all kinds of color-conversion display systems, including organic and inorganic phosphors.

## 1. Introduction

Recently, micro-light-emitting diodes (micro-LED) (with a chip size less than 100 μm) have begun to emerge as a potentially disruptive display technology, provided that their cost can be reduced significantly [1,2]. To produce full-color images, millions of red, green and blue (RGB) micro-LED chips from different semiconductor wafers (GaN for blue and green sub-pixels, and AlGaInP for red sub-pixels) should be transferred to the same display substrate, e.g., glass [3,4]. However, due to the complex manufacturing process (mass transfer technology) and driving circuit design (pulse width modulation), the cost of RGB micro-LED displays remains relatively high at the present time. To overcome these problems, a simpler method has been proposed that could achieve full color by assembling a blue micro-LED array with a color conversion layer [5,6,7]. The blue light from the micro-LED array pumps the color conversion materials, such as phosphors, quantum dots (QD), or perovskite nanocrystals, to obtain vivid colors. The quantum dot color filter (QDCF) on top of the micro-LED array is mainly fabricated by inkjet printing or photolithography [8,9]. In addition to the simpler fabrication process, another advantage of the color conversion micro-LED display is wide color gamut. A display with a wider color gamut can reproduce images more accurately. Rec. 2020, covering 99% of the Pointer’s gamut in the natural environment, has been widely used as a new standard for evaluating the color performance of display panels [10]. In order to obtain a high performance micro-LED display with wide color gamut, high color conversion efficiency (CCE) and large viewing angle, the following three main obstacles should be overcome: (1) blue light leakage of the red and green sub-pixels. If the color conversion material cannot completely convert the excitation light, the leaked blue light will seriously reduce the color purity of the display [11]. (2) The limitation of the external quantum efficiency (EQE) of the color conversion film. The EQE of a color conversion film can be defined as the ratio of the number of down-converted photons (red or green) to the number of incident photons (blue). Therefore, the EQE not only depends on the quantum yield of the color conversion material, but also on the light extraction efficiency of the color conversion film [12]. (3) Mismatched radiation patterns of the RGB sub-pixels. The isotropic emission characteristics of color conversion materials determine the angular spectrum of the red and green sub-pixels. However, if there is no scattering particle in the blue sub-pixel, the outgoing angular distribution is determined by the optical characteristics of the blue LED chip. Therefore, the angular spectrum mismatch from the RGB sub-pixels may cause a significant angular color shift [13,14].

An intuitive way to eliminate the blue light leakage is to increase the optical density of the QDCF. However, a high concentration QD film would exhibit following disadvantages: (1) poor dispersion ability, which results in a low quantum yield [15]. (2) Self-absorption may also lead to a lower optical conversion efficiency [16]. Different kinds of optical films or structures have been used to overcome these problems. A conventional method to prevent blue light leakage from the color conversion film is to laminate another pigment color filter array on top of it. As mentioned in [17], such a color filter array not only absorbs the leaked blue light, but also improves the ambient contrast ratio of the color-converted micro-LED display. However, the absorbed blue light is wasted. To improve the CCE of the QD film, Chen et al. proposed to deposit a distributed Bragg reflector (DBR) on top of the QD film [18]. Normally, at least five pairs of TiO_2_/SiO_2_ should be used in the DBR to achieve sufficient reflectivity in the blue spectral range while keeping a high transmittance for the green and red channels. The leaked blue light will be recycled by the DBR and then re-enter the color conversion film to excite the phosphors. As a result, a higher CCE can be obtained. However, if we consider the RGB sub-pixels together, the DBR should be patterned to only cover the red and green sub-pixel areas and avoid blocking the blue light in the blue sub-pixels. Although there are several designs that use patterned DBR in color-conversion micro-LED displays [19,20], commercial products are still lacking due to the high cost and complex manufacturing processes of patterned DBR.

In this paper, we propose a patterned cholesteric liquid crystal (CLC) polymer film to improve the display performance of the color-conversion micro-LED display. The proposed CLC film consists of two types of textures: focal conic texture (FC-CLC) and planar texture (P-CLC). The P-CLC on the red and green sub-pixels acts as a DBR to recycle the leaked blue light, while the FC-CLC on the blue sub-pixels functions as scattering particles to match the angular distribution with the R and G sub-pixels. In this experiment, we fabricated several patterned CLC textures with sizes ranging from 10 μm to 80 μm and measured their optical properties for further optimization simulation. In addition, we developed a hybrid QDCF comprising of cadmium (Cd)-based red QD and homemade green perovskite nanocrystals to achieve a wide color gamut [21]. According to our simulation results, the patterned CLC film with an optimized reflection band can not only recycle the leaked blue light, but also reduce the crosstalk between blue and green color filters. Therefore, our proposed patterned CLC polymer film improves the CCE of the display system by about 143%. In addition, since the FC-CLC on the blue sub-pixels helps to reduce the mismatched angular radiation patterns of RGB sub-pixels, the average angular color shift Δu’v’ is reduced from 0.03 to 0.018 at the 50° viewing angle where the color shift is largest. Finally, a proof-of-concept experiment was carried out to validate our optical simulation.

## 2. Materials and Methods

### 2.1. Patterned CLC Film

Our CLC film consists of patterned focal conic and planar textures. The P-CLC is formed when the CLC directors are along the helical axis, which is induced by the chiral dopant. When a circularly polarized light with the same handedness (e.g., right-handed circularly polarized (RCP)) is incident on the P-CLC film, the Bragg condition is satisfied, and the RCP light will be reflected. The central wavelength (*λ_o_*) of the Bragg reflection depends on the pitch length (*p*) and average refractive index (*n*) of the employed LC material as *λ_o_* = *p*⋅*n*. We intentionally designed the CLC film to have high reflectivity covering our blue LED emission band, while keeping high transmittance in the red and green spectral region. This type of CLC film can be applied to the red and green sub-pixels as a DBR for recycling the unabsorbed blue light. On the other hand, we purposely patterned the FC-CLC film on top of the blue sub-pixels. The FC-CLC is formed when the helical axis is randomly arranged. These FC-CLC segments scatter the blue micro-LED light to match the isotropic radiation pattern of green and red sub-pixels from the color conversion layer, such as quantum dots. As a result, the angular color shift caused by the mismatched RGB radiation pattern is minimized.

**Figure 1:**
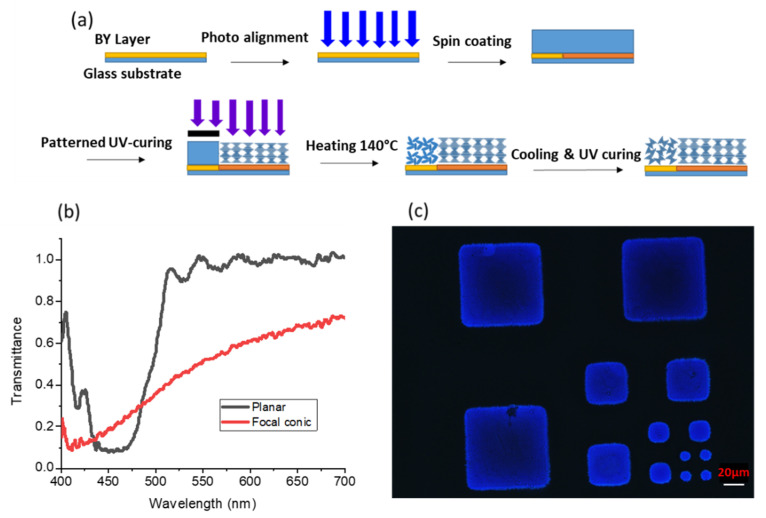
(**a**) Fabrication process of a patterned cholesteric liquid crystal (CLC) film. (**b**) Measured transmission spectra of planar and focal-conic texture CLC on a patterned CLC film. (**c**) Microscope image showing a patterned CLC film with 10 µm, 20 µm, 40 µm, and 80 µm feature sizes.

In this experiment, we found that it was rather difficult to fabricate the proposed patterned two-texture CLC film either by pattern curing or a photo-alignment process. This is because to form high quality Bragg reflection in the planar texture, surfactants are required to arrange the CLC molecules neatly on the interface between CLC material and air. However, due to the effect of the surfactants, most of the CLC molecules are partially aligned, so that the CLC film is similar to an imperfect planar texture, instead of a focal-conic texture [22]. To solve this problem, we developed a new fabrication process as Figure 1a illustrates. In this experiment, we prepared a CLC precursor consisting of 92.95 wt.% reactive mesogen RM257 (from HCCH, Beijing, China) as LC monomer, 2.8 wt.% S5011 or R5011 (from HCCH, Beijing, China) chiral dopants, 4 wt.% of photo-initiator Irgacure 651 (from BASF, Ludwigshafen, Germany), and 0.25 wt.% of surfactant Zonyl 8857A (from DuPont, Wilmington, DE, USA). First, we spin-coated a thin brilliant yellow (BY) photo-alignment layer on a cleaned glass substrate and cured it with an ultraviolet (UV) light. Next, we spin-coated the CLC precursor on top of the BY layer to replicate the alignment. In our design, the helical pitch length of the CLC is about 298 nm to reflect blue light, and the thickness of film is ∼8 helical pitches to establish Bragg reflection with a reflectivity larger than 90%. To create two different textures on the CLC film, we used a patterned photomask during UV curing. The photo-polymerization process took place only in the transparent area to form a stable polymer film (P-CLC). After that, the sample was heated to 140 °C, and the CLC material in the unexposed area (FC-CLC) became isotropic. Finally, the cooling process and UV curing were performed at the same time. During cooling, the liquid crystal transitioned from isotropic texture back to cholesteric, and the arrangement was completely random. We have successfully fabricated two different types of CLC films (planar and focal conic textures) on the same substrate. Figure 1b depicts the transmission spectra of the planar and focal conic textures of the film measured by a circularly polarized light with same handedness as the CLC film. In addition, several patterned CLC films with different feature sizes were fabricated to prove that they can satisfy different display applications. Figure 1c shows a micrograph of the patterned CLC film with 10 µm, 20 µm, 40 µm, and 80 µm feature sizes.

**Figure 2:**
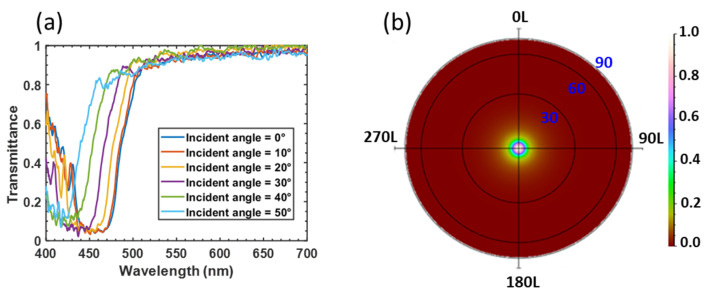
(**a**) Measured reflection spectrum of planar texture (P-CLC) film at different incident angles. (**b**) Measured angular transmission profile of focal conic texture (FC-CLC) film at normal incidence.

We further measured the optical properties of patterned CLC film for optical simulation, which will be described later. Figure 2a shows the measured reflection spectra of the P-CLC film composed of two CLC films with opposite handedness, while the incident unpolarized light’s angle varies from 0° to 50° at 10° intervals. As the incident angle exceeds 50°, due to blue shift, the reflectivity of P-CLC at the blue LED emission wavelength becomes negligible. Therefore, we can assume the transmittance of P-CLC film is nearly 100% at an incident angle greater than 60°. On the other hand, in the FC-CLC segment, the tiny multi-domain CLCs with random distribution scatter the incident light. The total transmittance is about 95%, and its angular transmission profile is plotted in Figure 2b. Unlike the reflection band of P-CLC, which exhibits a strong polarization selectivity, the scattering property of FC-CLC is polarization independent.

### 2.2. Device Modeling

**Figure 3:**
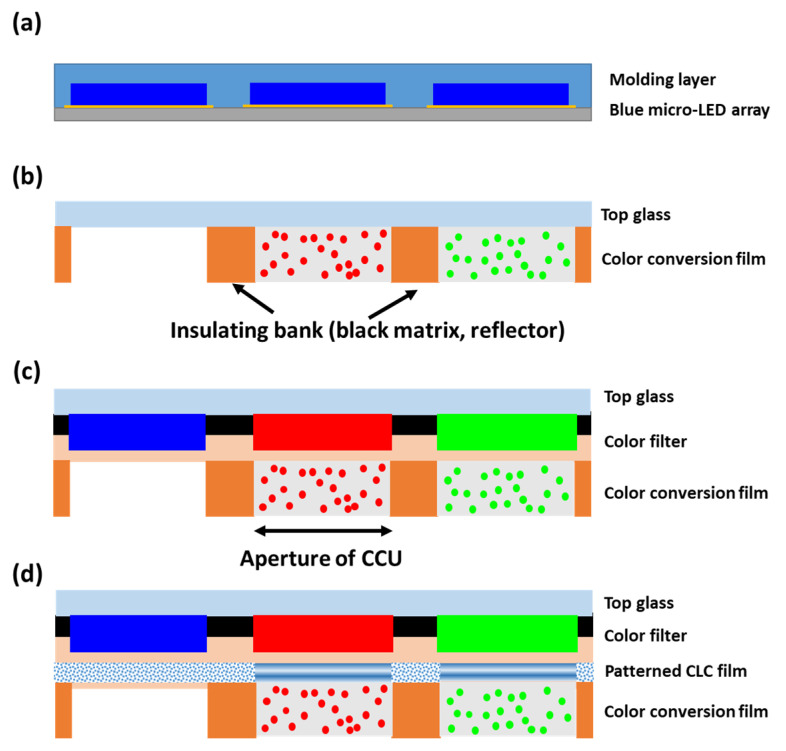
(**a**) Schematic diagram of the blue micro-light-emitting diode (LED) array, (**b**) the color conversion unit (CCU) consists of color conversion film only. (**c**) CCU consists of a color filter laminated on top of the color conversion film (control display system). (**d**) CCU consists of a color filter and a patterned CLC film laminated on the color conversion film (proposed display system).

The color-converted micro-LED display consists of two main parts. One is the narrow band blue LED array shown in Figure 3a. The blue LED array is protected and planarized by molding materials. A reflector with 90% reflectivity at the bottom of the LED chip would reflect the emitted light upward. The other part is a color conversion unit (CCU). In this paper, we investigate three types of CCU as shown in Figure 3b–d. The aperture of CCU is firstly set to be same as the LED chip size. Here, we first define the air gap between the layers of the device structure, which may have a significant impact on the display performance. In our model, the air gap only exists between the blue LED array and CCU. The refraction at the interface between CCU and the air narrows the angular distribution of input blue light, which improves the recycling of leaked blue light by the patterned CLC film. However, the air gap also reduces the light extraction efficiency of the micro-LED array. The influence of the air gap will be evaluated in the discussion paragraph.

Through the device structure shown in Figure 3b, the CCE and blue light leakage rates of hybrid QDCF with different optical densities are analyzed. Although high QD concentration helps prevent blue light leakage, the strong self-absorption in the color conversion film leads to a decreased CCE. The mechanisms will be discussed later. Figure 3c shows the device structure of a conventional color-conversion micro-LED display. The color filter is aligned with the RGB sub-pixels to absorb the leaked blue light. This device is used as a control panel for evaluating the improvements that occur due to adding a patterned CLC to the display system, as shown in Figure 3d. The proposed patterned CLC film exhibits two attractive features: (1) compared to a DBR film, its low cost and simple fabrication process is more friendly to mass production. (2) Two kinds of optical characteristics can be realized on the same substrate: the P-CLC on the red and green sub-pixels acts as a DBR to recycle the leaked blue light, and the FC-CLC on the blue sub-pixel functions as scattering particles to diverge the angular spectrum to match the green and red sub-pixels. Here, the CLC films with opposite handedness are assembled to recover the unpolarized leakage light from the blue micro-LED. The top color filter array is still needed to reduce the excitation of ambient light.

**Figure 4:**
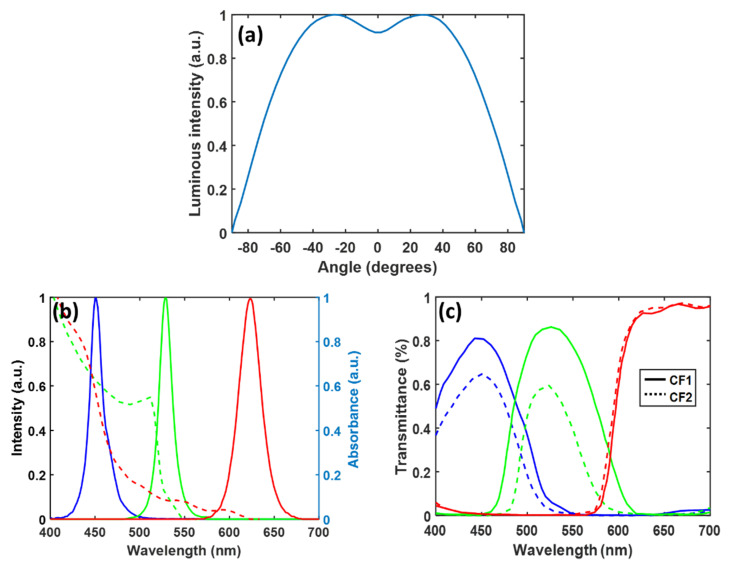
(**a**) Simulated radiation pattern of the micro-LED array (chip size 100 μm × 100 μm). (**b**) Emission spectrum of the blue micro-LED; absorbance (dashed lines) and photoluminescence spectra of green perovskite nanocrystal and red QD. (**c**) Transmission spectra of color filters CF1 and CF2.

Next, we use raytracing software (LightTools, 8.7.0, Synopsys Inc., Santa Clara, CA, USA) to simulate the performance of our proposed display system. The size of the LED chip used in our simulation model is 100 μm × 100 μm, and Table 1 lists the material properties of the flip-chip blue LED at three specified wavelengths. The radiation pattern of blue micro-LED emitted from the molding layer is plotted in Figure 4a.

Figure 4b depicts the emission spectrum of the blue LED. The photoluminescent characteristics of QD are simulated by Advanced Physics Module in LightTools. The absorption and emission spectra of green perovskite nanocrystals and cadmium-based red QDs are also plotted in Figure 4b [23,24]. In our model, the mean free path, which is the average length of ray propagation before impacting QD particles, is used to represent the concentration of the color conversion film [25]. Due to limitations in the manufacturing process [8,26], the thickness of color conversion film is about 5–9 µm. The measured photoluminescence quantum yield (PLQY) of our perovskite film is 0.7, and the refractive index of the color conversion film is 1.5. In the raytracing mode, the emitted blue light from micro-LED enters the hybrid QDCF and a portion of the blue light is down-converted to red or green light with isotropic radiation, and the unabsorbed light will continue to propagate without changing its path. In addition, to prevent color crosstalk between each pixel, a black matrix is applied in the sidewall between each sub-pixel and the bottom reflector, which has a reflectivity of 90% to recycle light emitted backwards. Two types of color filters (CFs) are used in our simulation, and their transmission spectra are shown in Figure 4c. Compared to CF1, CF2 provides a wider color gamut, but the tradeoff is lower light efficiency. Meanwhile, we also apply the measured optical properties of the patterned CLC film to our LightTools model to simulate its performance in the display system.

## 3. Results

### 3.1. Tradeoff in High Concentration QD Film

**Figure 5:**
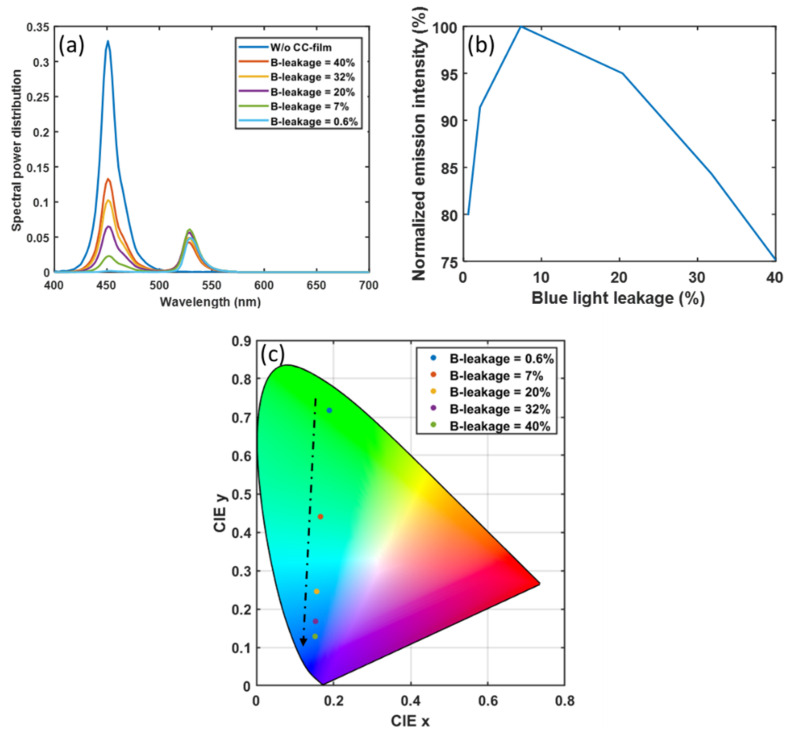
Impact of leaked blue light on (**a**) the spectral power distribution in the green sub-pixels, (**b**) the intensity of down-converted green light, and (**c**) the corresponding color coordinates in CIE 1931.

As mentioned above, a high concentration color-conversion film with a short mean free path helps reduce the blue light leakage. Here, we plot the relationship between the spectral power distribution in the green sub-pixel in Figure 5a. The spectrum consists of two parts: one is converted green light, and the other is unabsorbed blue light. As the concentration of perovskite nanocrystals increases, more blue light will be converted to green light. Thus, the intensity of the down-converted light first increases, and the blue light leakage decreases. However, if the concentration of perovskite nanocrystals is further increased, the down-converted green light will suffer from more severe self-absorption, and the intensity of the output green light will decrease. The normalized intensity of down-converted green light as a function of blue light leakage (blue light intensity of device structure with different concentration CC films divided by blue light intensity of device structure without CC film) is shown in Figure 5b. In addition, the color performance of the display is also affected by the leaked blue light. Based on the emission spectrum in Figure 5a, the corresponding color coordinates in the CIE 1931 color space are plotted in Figure 5c. To reduce the impact of unabsorbed blue light in the green sub-pixels on color purity, the proportion of blue light leakage should be less than 0.6%. However, under such conditions, the green light intensity would drop by about 20%. In addition, a high concentration of color conversion film also means more material consumption and higher cost. The peak external EQE (ratio of blue photons incident in CCU to green photons converted from CCU) is 18.16% corresponding to 7% of blue light leakage.

### 3.2. Performance Improvement by the Patterned CLC

In practical display applications, the color-conversion film may be excited by ambient light, which would severely degrade the image quality. Therefore, as Figure 3b shows, the color filter array should be aligned with the RGB sub-pixels. This is our control display system. On the other hand, our proposed display system with a patterned CLC film is shown in Figure 3c. In the following, we evaluate the improvement of our proposed patterned CLC film system versus the control system in three aspects: (1) color conversion efficiency, (2) color gamut, and (3) viewing angle.

#### 3.2.1. Color Conversion Efficiency

**Figure 6:**
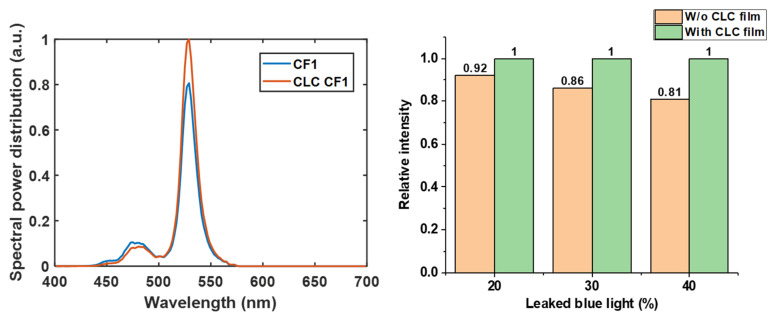
(**a**) Comparison of the spectral power distribution of green sub-pixel between display systems with and without a P-CLC film. (**b**) The CCE improvement by P-CLC under display system with different blue light leakage rates.

In the color conversion micro-LED display system, the power efficiency largely depends on the CCE of the color conversion film. As described above, the P-CLC film can reflect the leaked blue light back to the color conversion film, thereby increasing CCE. Generally, the more blue light leakage, the larger the improvement of the P-CLC film. Therefore, it is important to set the blue light leakage rate close to a practical value. When a QDCF is fabricated by the inkjet printing method, the uneven morphology, called “coffee ring”, can cause severe blue light leakage (about 80%) in areas where the material is thinner. By employing polymer-based inks, a uniform morphology can be achieved, and the blue light leakage is improved to about 20% [27]. Another method for fabricating a QDCF is photolithography. However, due to limited film thickness, the blue light leakage is about 40% when the film thickness is 5 µm [28]. From the above discussion, we find that the blue light leakage rate for most QDCF is about 20% to 40%. Therefore, in the simulation model, we used these numbers to analyze the CCE enhancement due to our patterned CLC film. Figure 6a compares the spectral power distribution between the two display systems with and without a patterned CLC film, when the blue light leakage is 40%. In addition, we plot the increased CCE of the laminated CLC film as a function of blue light leakage in Figure 6b. Obviously, using P-CLC for display systems with greater blue light leakage can achieve a higher CCE improvement. Generally, we can improve the CCE by about 20% through our patterned CLC film.

#### 3.2.2. Color Gamut

**Figure 7:**
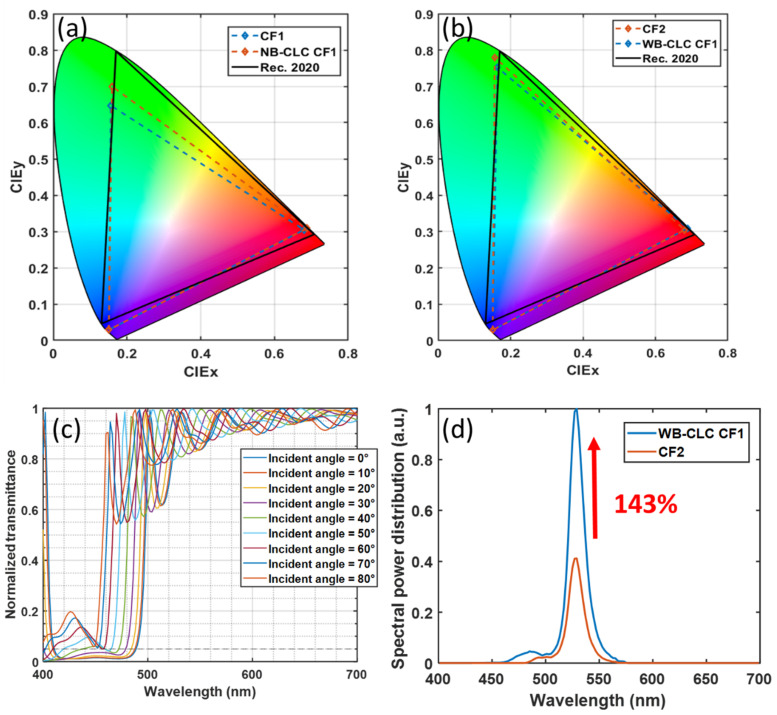
(**a**) Simulated color gamut for display systems with CF1 only, and with CF1 and narrowband CLC film. (**b**) Simulated color gamut for display systems with CF2 only, and with CF1 and wideband CLC film. (**c**) Reflection spectra of planar texture wide band CLC (WB-CLC) film at different incident angles. (**d**) Comparison of the spectral power distribution of green sub-pixel between display systems with CF2 only, and with CF1 and wideband CLC film.

Vivid color is a highly desirable requirement of all displays. The narrow emission spectra of green perovskite nanocrystals and red QD materials help provide a wide color gamut. However, the leaked blue light may reduce the color purity, thereby shrinking the color gamut. Here, based on the same assumption, in Figure 7a we plot the simulated color gamut of the two display systems in CIE 1931 color space when the blue light leakage rate is 40%. As Figure 6a shows, the CLC film can recycle the leaked blue light to excite more green light and reduce the light intensity of the leaked blue light in the spectral range from 450 nm to 500 nm. As a result, the color gamut of the display system with CLC film is extended from 73.5% to 81.34% coverage of Rec. 2020. However, such a color gamut is smaller than expected, mainly caused by the crosstalk between the blue and green color filters. Some of the blue light can pass through the green filter in the 460–500 nm range, thereby reducing the color purity. To solve this problem, a color filter (CF2) with less crosstalk between the blue and green channels can be applied to widen the color gamut [29,30]. Compared to CF1, CF2 can extend the color gamut from 73.5% to 89.4% of Rec. 2020, as shown in Figure 7a,b. However, the average transmittance of CF2 in the emission spectrum of perovskite nanocrystals is only 54.5%. Such a low transmittance greatly reduces the CCE of the display system. To overcome this problem, we proposed to employ a high-transmittance color filter (CF1) with a wide band CLC film (WB-CLC film). Because the reflection bandwidth of the CLC film is determined by the product of pitch length and LC birefringence (∆n), the WB-CLC films can be fabricated by using a high ∆n LC monomer, e.g., Merck RMS16/091, ∆n = 0.3 at 450 nm. The simulated reflection bands of the WB-CLC film at different incident angles are shown in Figure 7c. In order to identify two types of CLC films clearly, we named the experimental CLC film with lower birefringence as narrow band CLC film (NB-CLC film), and the newly proposed CLC film with higher birefringence as wide band (WB) CLC film. The high reflectivity of WB-CLC film in the 450–490 nm spectral range can significantly reduce the blue light leakage in the blue-green crosstalk region of CF1. As a result, WB-CLC film assembled with CF1 can also achieve a wide color gamut, which covers 88.2% of Rec. 2020. In addition, both CF1 and WB-CLC films have high transmittance in the green spectral range, as compared to CF2, our newly proposed device structure can achieve the same color gamut, but with 143% higher CCE, as Figure 7d shows.

#### 3.2.3. Viewing Angle

**Figure 8:**
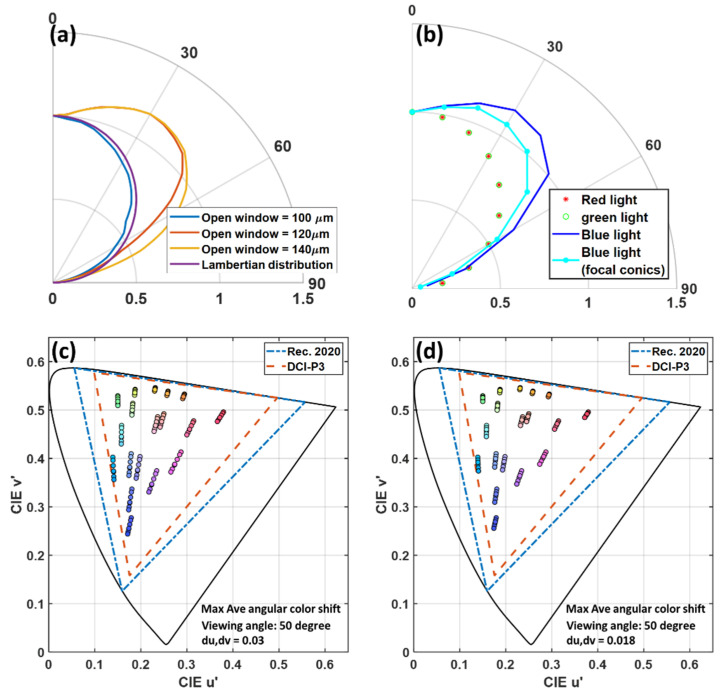
(**a**) Angular intensity distribution of the blue sub-pixels of different aperture size color conversion units. (**b**) Angular intensity distribution of red, green, and blue (RGB) sub-pixels (aperture size = 120 µm). Simulated color shift of the first 18 colors in Macbeth ColorChecker from 0° to 60° viewing angle for color conversion micro-LED display (**c**) without FC-CLC film and (**d**) with FC-CLC film.

In practical applications, viewers may view the display from different angles, so the display is required to have a significant angular color shift. Different from the isotropic emission of QD particles in the green and red sub-pixels, the angular distribution of the blue sub-pixels is mainly determined by the emission spectrum of the micro-LED and the device structure of the color conversion unit. In the above discussion, the aperture size of the CCU is the same as the LED chip size. Therefore, most of the sidewall emission of micro-LED is absorbed by the insulating bank, so that the radiation pattern of the blue sub-pixel is close to Lambertian distribution. However, in practical applications, the CCU aperture size can be larger than the LED chip size to collect the sidewall emission from the micro-LED. As a result, the optical efficiency is improved. As the aperture increases, more sidewall emissions are extracted, and the radiation pattern becomes closer to the batwing distribution. In Figure 8a, the radiation pattern of blue sub-pixels of the CCU with different aperture sizes is shown. Even the radiation pattern of the blue sub-pixel in the small aperture size matches the angular distribution (Lambertian distribution) of the RG sub-pixels, its optical efficiency is only 74% and 65% as compared to the CCU with 120 μm and 140 μm apertures. Therefore, in order to maintain high optical efficiency, a larger aperture size is preferred.

Generally, scattering particles are added in the blue channels to match the angular distribution with the RG sub-pixels. Here, we use the FC-CLC film as a scattering medium to replace the scattering particles. As shown in Figure 8b, as the aperture of CCU is 120 µm, our FC-CLC film on the blue sub-pixels helps to reduce the mismatch with the green and red sub-pixels. Here, we use the first 18 colors in Macbeth ColorChecker to evaluate the angular color shift of the color conversion micro-LED display, as shown in Figure 8c,d. At the 50° viewing angle, the most severe angular color shift occurs. By using the FC-CLC film, under the same conditions, the average color shift Δu’v’ of the 18 reference colors is reduced from 0.03 to 0.018. In addition, in the most severe color shift channel (medium red), Δu’v’ is decreased from 0.047 to 0.029.

The CLC film’s thickness limits the scattering properties of the focal conic texture. A better fabrication process can further increase the film’s thickness, so a display system with less angular color shift can be obtained.

### 3.3. Proof-of-Concept Experiment

**Figure 9:**
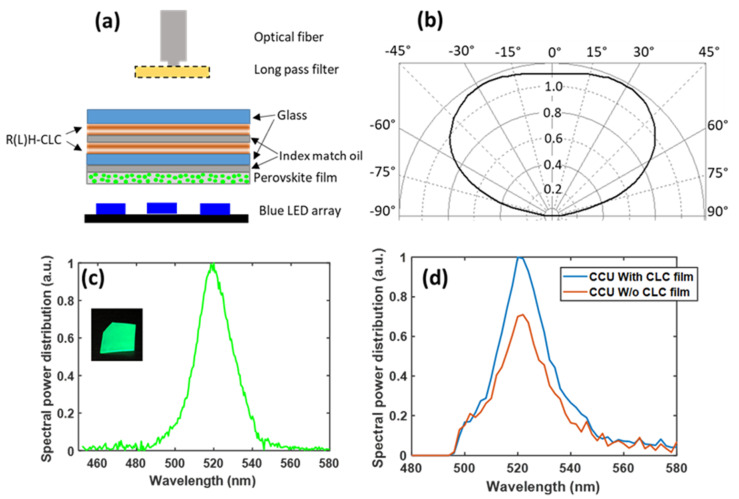
(**a**) Schematic diagram of the experimental setup. (**b**) Measured radiation pattern of the LED array. (**c**) Measured emission spectrum of the CsPbBr_3_ perovskite film. Inset: CsPbBr_3_ perovskite film under 365 nm UV light. (**d**) Measured emission spectrum of down-converted green light with and without the CLC film (filtered by the long pass filter with 500 nm cutoff wavelength).

We also conducted a proof-of-concept experiment, as shown in Figure 9a. We used a blue LED array with a central wavelength around 450 nm as the emission source. The radiation pattern of the blue LED array is measured by a goniophotometer (RiGO801 Techno Team Vision) as shown in Figure 9b. The CsPbBr_3_-polystyrene perovskite-polymer composite film was prepared by the swelling-deswelling microencapsulation method [31]. Its emission spectrum is shown in Figure 9c. The central wavelength is 520 nm and the full width at half maximum (FWHM) is 21 nm. In this experiment, the CCU is composed of two CLC films with opposite handedness and the CsPbBr_3_-polystyrene perovskite–polymer composite film. The index matching oil is applied between each layer of the CCU. In addition, the blue LED array is covered by the molding material, and there is no optical contact between the CCU and blue LED array. The blue light leakage ratio is about 60%. To evaluate the effectiveness of the CLC film, we prepared a control measurement using glass substrates as a reference, and then conducted an experimental test with a two-layer CLC films with opposite handedness. After the long pass filter (cutoff wavelength is 500 nm), the normalized emission spectrum received by the fiber-optic spectrometer (Ocean Optics HR2000CG-UV-NIR) from the device structures with and without CLC film is plotted in Figure 9d. Note that the optical fiber can only receive the light emitted perpendicular to the perovskite film. However, the emission of the perovskite nanocrystals is isotropic, and the ratio of the intensity improvement should be the same at all angles. Due to the limitation of our fabrication facility, in this experiment, the micro-LED array is replaced by an LED array, and some parts of the device structure in the CCU, such as insulating banks, are not considered. In addition, the backward reflector (R = 90%) at the bottom of the micro-LED array was not applied in the experiment. All these factors will affect the absolute improvement of CCE by adding the CLC film. However, in this proof-of-concept experiment, we still observed that by assembling CLC films with opposite hands, blue light leakage can be greatly reduced, and the CCE of the color conversion micro-LED display system can be improved.

## 4. Discussion

**Figure 10:**
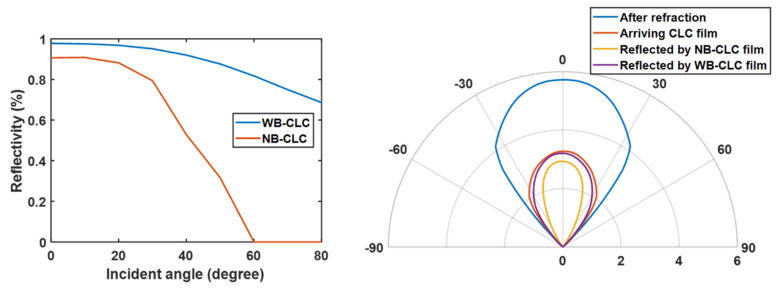
(**a**) Average reflectivity of WB and NB P-CLC films in the blue light spectrum (micro-LED) at different incident angles (in air). (**b**) Angular intensity distribution of blue light in the RG sub-pixels at different layers of the color-converted micro-LED.

In this paper, we apply both WB- and NB-CLC films to the CCU for recycling leaked blue light. According to the reflection spectra of the WB-CLC and NB-CLC films in Figure 7a,c, the average reflectivity of the P-CLC film in the spectrum range of blue light can be calculated by:

(1) $$R_{\theta }=\frac{\int S_{LED}(\lambda )\times R_{CLC}(\lambda ,\theta )d\lambda }{\int S_{LED}(\lambda )d\lambda }$$

where *S*_LED_ is the spectral power density of blue light emitted by the micro-LED, and R_CLC_ is the reflection spectra of CLC film at different incident angles. Based on Equation (1), the average reflectivity of the WB- and NB-CLC films as a function of incident angles is plotted in Figure 10a. The refraction between the air gap between the micro-LED array and QD film narrows the angular distribution of blue light inside the CCU. As shown in Figure 10b, the angular distribution of blue light inside the CCU is limited to ±41°. After being absorbed by the color conversion material, the angular distribution of blue light reaching the interface of the color conversion film and CLC film is also plotted in Figure 10b. In order to specifically analyze the blue light recycled by the CLC film, the angular distribution of the blue light reflected by the WB- and NB-CLC films is also shown in Figure 10b. Since the average reflectivity of the NB-CLC film decreases at large incident angles, blue light with an incident angle greater than 20° in the CCU (*n* = 1.5) cannot be effectively recycled by the CLC film. On the other hand, using WB-CLC film with high reflectivity in large incident angles can recycle most of the blue light. Through the above discussion, as long as the reflection band of the CLC film is optimized, the patterned CLC film can recycle most of the leaked blue light, thereby improving the CCE of the color conversion micro-LED display.

There is still a need to overcome the low light extraction efficiency of the micro-LED array caused by the air gap between the LED molding layer and the CCU. The use of a microlens array or sidewall reflectors to collimate the LED emission [32,33] has been widely used to reduce the total internal reflection (TIR) at the interface between the molded layer and air, thereby increasing the light extraction efficiency. In addition, because in the green and red sub-pixels the leaked blue light will be recycled or absorbed by the CLC film and the color filter, the collimated blue LED emission will not affect the angular color shift of the display.

## 5. Conclusions

We have fabricated patterned CLC films with two different optical functionalities (reflection and scattering) on the same substrate, with the feature sizes ranging from 10 µm to 80 µm. In addition, the green perovskite–polymer composite film is also demonstrated. The optical properties of the CLC film and the perovskite–polymer composite film are further applied to the optical simulation model to evaluate the performance of our proposed display system. According to our simulation results, the patterned CLC film not only improves the CCE by recycling leaked blue light, but also alleviates the crosstalk between green and blue color filters. Therefore, compared to the display system with color filters only, the patterned CLC film can increase the CCE by 143% (under ~90% Rec. 2020). In addition, the FC-CLC film scatters the emission light from micro-LED in blue sub-pixels to reduce the radiation pattern mismatch with the green and red sub-pixels, thereby reducing the average color shift from 0.03 to 0.018 at the viewing angle with the most severe angular color shift. We also conducted a proof-of-concept experiment and verified that the patterned CLC film improves the CCE of the color conversion film by about 43% (under 60% blue light leakage). Finally, this kind of patterned CLC film is applicable to advancing all kinds of color-conversion display systems, including organic and inorganic phosphors.

## Figures and Tables

**Table 1:** Material parameters used in the blue micro-LED array.

Material Parameters	630 nm		520 nm		465 nm	
	*n*	*k*	*n*	*k*	*n*	*k*
Molding layer	1.48	0	1.49	0	1.50	0
Blue LED chip	2.34	4 × 10^−5^	2.38	4 × 10^−5^	2.44	4 × 10^−5^
Glass substrate	1.5	0	1.5	0	1.5	0
